# Early activating somatic *PIK3CA* mutations promote ectopic muscle development and upper limb overgrowth

**DOI:** 10.1111/cge.13543

**Published:** 2019-05-09

**Authors:** Sofia Frisk, Fulya Taylan, Izabela Blaszczyk, Inger Nennesmo, Göran Annerén, Bettina Herm, Eva‐Lena Stattin, Vasilios Zachariadis, Anna Lindstrand, Bianca Tesi, Tobias Laurell, Ann Nordgren

**Affiliations:** ^1^ Department of Molecular Medicine and Surgery, Center for Molecular Medicine Karolinska Institutet Stockholm Sweden; ^2^ Department of Clinical Genetics, Karolinska University Laboratory Karolinska University Hospital Stockholm Sweden; ^3^ Department of Surgical and Perioperative Sciences, Hand and Plastic Surgery Umeå University Hospital Umeå Sweden; ^4^ Department of Pathology Karolinska University Hospital Stockholm Sweden; ^5^ Science for Life Laboratory, Department of Immunology, Genetics and Pathology Uppsala University Uppsala Sweden; ^6^ Child and Adolescent Habilitation Centre Östersunds Hospital Östersund Sweden

**Keywords:** accessory, cell fate, ectopic, muscle hypertrophy, muscular hypertrophy, *PIK3CA*, PROS

## Abstract

*PIK3CA*‐related overgrowth spectrum is a group of rare genetic disorders with asymmetric overgrowth caused by somatic mosaic *PIK3CA* mutations. Here, we report clinical data and molecular findings from two patients with congenital muscular upper limb overgrowth and aberrant anatomy. During debulking surgery, numerous ectopic muscles were found in the upper limbs of the patients. DNA sequencing, followed by digital polymerase chain reaction, was performed on DNA extracted from biopsies from hypertrophic ectopic muscles and identified the somatic mosaic *PIK3CA* hotspot mutations c.3140A > G, p.(His1047Arg) and c.1624G > A, p.(Glu542Lys) in a male (patient 1) and a female (patient 2) patient, respectively. Patient 1 had four ectopic muscles and unilateral isolated muscular overgrowth while patient 2 had 13 ectopic muscles and bilateral isolated muscular overgrowth of both upper limbs, indicating that her mutation occurred at early pre‐somitic mesoderm state. The finding of *PIK3CA* mutations in ectopic muscles highlights the importance of *PIK3CA* in cell fate in early human embryonic development. Moreover, our findings provide evidence that the disease phenotype depends on the timing of *PIK3CA* mutagenesis during embryogenesis and confirm the diagnostic entity *PIK3CA*‐related muscular overgrowth with ectopic accessory muscles.

## Graphical abstract

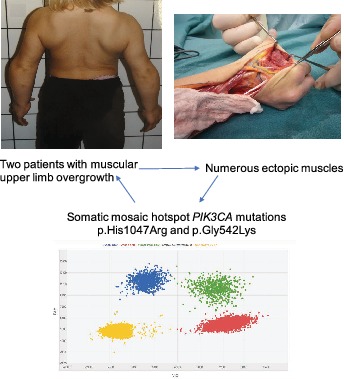

## 1 INTRODUCTION

Activating somatic mutations in the phosphatidylinositol‐4,5‐bisphospate 3‐kinase, catalytic subunit alpha gene (*PIK3CA*) occur frequently in congenital overgrowth syndromes and in human cancer.^1^ *PIK3CA* encodes the p110 α catalytic subunit of phosphoinositide 3‐kinase (PI3K), that phosphorylates phosphatidylinositol to generate phosphatidylinositol 3,4,5‐trisphosphate (PIP3). PIP3 plays a key role in activating signaling cascades including inhibition of apoptosis, activation of protein synthesis, and enhanced cell survival.^2^

The umbrella term *PIK3CA*‐related overgrowth spectrum (PROS) is a heterogeneous group of rare genetic disorders with overgrowth caused by somatic mosaic *PIK3CA* mutations.^3^ The variable expression of symptoms within PROS is mainly explained by the timing and location of the initiating *PIK3CA* mutation, but the reason behind the high degree of interindividual phenotypic heterogeneity is unknown.^4^ Diseases within PROS are classified on the basis on anatomical differences, for example, isolated macrodactyly (OMIM 155500), megalencephaly‐capillary malformation‐polymicrogyria syndrome (OMIM 602501), and congenital lipomatous asymmetric overgrowth of the trunk with lymphatic, capillary, venous, and combined‐type vascular malformations, epidermal nevi, scoliosis/skeletal and spinal anomalies (OMIM 612918).

The terminology “aberrant muscle syndrome” or “accessory muscle syndrome” has been suggested to describe patients with “hypertrophy of the hand and arm because of aberrant muscles with or without hypertrophy of the muscles.”^5^ It has been proposed that an increased number of neuromuscular junctions and a change in the muscle‐tendon ratio is involved in muscle hypertrophy development.^6^ The etiology remains still undetermined but it has recently been reported that isolated congenital muscular upper limb overgrowth can be related to somatic mosaic *PIK3CA* mutations.^7^, ^8^

Somatic activating *PIK3CA* mutations are common in at least 12 different cancer types.^1^, ^9^, ^10^, ^11^ Previous studies show that the same *PIK3CA* hotspot mutations found in cancer are found in PROS.^12^, ^13^, ^14^, ^15^, ^16^ To date, the only malignancy that has been reported in the 419 known individuals with PROS is Wilms tumor (OMIM 194070), which has been described in 12 individuals (2.86%); six children with a confirmed molecular diagnosis^12^, ^17^, ^18^, ^19^, ^20^, ^21^ and six children where genetic testing has not been performed.^22^, ^23^, ^24^, ^25^, ^26^ It has recently been suggested to use monitoring of cell‐free DNA in urine to screen for renal involvement in PROS.^27^

Although it is critical for normal cell growth and survival, the role of *PIK3CA* in early human development is poorly characterized. Because not all cells in overgrown tissues in PROS appear to have *PIK3CA* mutation, it has been suggested that *PIK3CA* mutation‐positive cells exert growth‐promoting effects on adjacent or distant cells.^4^ In cells with strong activation of PI3K signaling pathway, *PIK3CA* mutations may lead to lineage‐specific cell loss during or after differentiation because of mechanisms such as oncogene‐induced senescence.^28^ Previous studies have highlighted that PI3K signaling is crucial for embryonic development and plays an important role in the control of pluripotency and differentiation.^29^ Recent studies have also highlighted a prominent role for PI3K signaling during cell differentiation and determination of cell fate in mouse models.^30^, ^31^

In this study, we confirm a novel PROS phenotype with isolated muscle overgrowth and ectopic muscles and show evidence that *PIK3CA* regulates cell fate in early human embryonic development.

## 2 MATERIAL AND METHODS

### 2.1 Patients and ethical approvals

We included two patients with local muscular overgrowth of the upper extremity that were seen at the Department of Clinical Genetics, Karolinska University Hospital, Stockholm. The study was performed in accordance with the Declaration of Helsinki, and the local ethical board in Stockholm approved the study. Informed consents were obtained from each participating individual or their legal guardians according to local ethical guidelines and the parents have approved publication of clinical data and pictures.

### 2.2 Oncomine solid tumor panel

DNA from patient 1 was extracted from paraffin‐embedded tissue containing pathological lesions from dorsal ectopic muscle of the hand. We used the Ion Torrent Oncomine Solid Tumor DNA Panel kit, on the Ion Torrent S5 (Thermo Fisher Scientific, Massachusetts) according to the manufacturer's instructions. The analyzed genes were *KRAS, BRAF, EGFR, NRAS* and *PIK3CA* (NM_006218.1).

### 2.3 Whole genome sequencing

Whole genome sequencing (WGS) was performed on DNA extracted from affected tissue and blood from patient 2 together with blood samples from the healthy parents. Libraries for sequencing on Illumina HiSeq X (Illumina Inc, San Diego, California) were prepared from genomic DNA using the Illumina TruSeq polymerase chain reaction (PCR)‐free kit with a mean insert size of >350 base pairs. This resulted in an average of 798 million mapped unique sequences per sample (range 737‐932 million) with a mean coverage of 38× (range 35‐44×). Reading mapping and somatic variant calling was performed with SpeedSeq framework 0.1.0, using reference genome build GRCh37/hg19.^32^ All *PIK3CA* variants were explored with GEMINI^33^ and visualized in the integrative genomics viewer.^34^

### 2.4 Digital polymerase chain reaction

Fifty nanograms of genomic DNA extracted from affected muscle tissue from patients 1 and 2 was amplified with 1X QuantStudio 3D Digital PCR Master mix (Applied Biosystems, California) and commercially available TaqMan assay for *PIK3CA* p.(His1047Arg) and p.(Glu542Lys) mutation (Applied Biosystems, California), according to the manufacturer's instructions. Extracted DNA was quantified using Qubit fluorometry (Thermo Fisher Scientific, Massachusetts). Fifteen microliters of PCR reaction mixes were loaded into QS3D Digital 20 K V2 chips (Applied Biosystems, California), which were then placed into GeneAmp PCR System 9700 thermocycler (Thermo Fisher Scientific). The chips were incubated at 96°C for 10 minutes, followed by 39 cycles at 54°C for 2 minutes and 98°C for 30 seconds. PCR was completed with a final incubation at 60°C for 2 minutes. After completion of PCR, each chip was scanned using QuantStudio 3D reader (Applied Biosystems). Digital PCR (dPCR) data were analyzed using PoissonPlus algorithm (version 4.4.10) with a confidence interval of 95% and the desired precision of 10% by QuantStudio 3D AnalysisSuite (version 3.1.2‐PRC‐build‐03). Using the two‐dimensional scatter plots, partitions for positive and negative amplification signals were classified for target and reference sequences using 6‐carboxyfluorescein and 2′‐chloro‐7′phenyl‐1,4‐dichloro‐6‐carboxy‐fluorescein channels.

### 2.5 Pathology

**Patient 1:** When the patient was 3 years old, macroscopic ectopic muscle tissue was removed from the dorsal part of the left hand. Hematoxylin‐eosin (H‐E) stained frozen sections and formalin fixed paraffin embedded (FFPE) sections stained with H‐E and van Gieson as well as with antibodies against p62 were analyzed.

**Patient 2:** Biopsies from ectopic dorsal interosseous muscle (I) and ectopic extensor digitorum muscle (II) were taken from the right side when the patient was almost 11 years old. H‐E stained sections from FFPE tissue were available for microscopic evaluation.

## 3 RESULTS

### 3.1 Clinical findings

**Figure 1:**
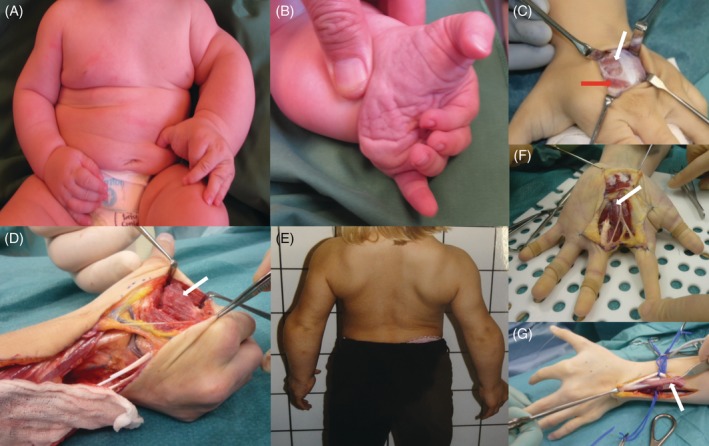
Clinical findings in patients. A, B, Overgrowth of left arm and hand with characteristic ulnar deviation of the index finger and adducted thumb in patient 1. C**,** White arrow shows atrophic ectopic muscle mass on the dorsum of left hand in patient 1. Red arrow shows normal junctura replaced by ectopic muscle. D**,** White arrow shows ectopic adductor muscle in patient 2. E**,** Bilateral overgrowth of shoulders, arms and hands in patient 2. F, White arrow shows ectopic palmar muscles extending proximally into carpal tunnel. G**,** White arrow shows ectopic extensor muscle dorsal to normal extensor digiti communis tendons and muscle

**Patient 1**: The 4‐year‐old male patient was born as the second child to healthy, non‐consanguineous parents. Pregnancy and delivery were normal and he was born at 40 + 4 weeks of gestational age. His birth length was 49 cm and his weight was 3780 g. Hemihypertrophy of the left arm and hand was noted directly after birth (Figure 1A). Magnetic resonance imaging and ultrasound of the left hand showed thickened muscles between metacarpals I and II and enlarged tendon to extensor pollicis brevis. Also, hypertrophy of extensor pollicis brevis and adductor pollicis longus muscles was noted. X‐ray showed widening of the metacarpals in a fan‐shaped matter. Ulnar deviation of the second to fifth finger was seen. A swan neck like deformity of the second finger was interpreted to be caused by intrinsic tightness. The thumb was abducted and hyperextended in the carpometacarpal joint (Figure 1B). The functional grip of the affected hand adapted well despite the malformations. During growth, a mass on the dorsum of his hand and a subcutaneous string in the volar forearm developed slowly and were removed surgically (Figure 1C). Operative exploration showed at least four different ectopic muscles of the hand and forearm (Table 1).

**Table 1:** Summary of molecular findings and phenotype in patients 1 and 2

Patient	1	2
Sex	M	F
*PIK3CA* mutation	c.3140A > G	c.1624G > A
Protein alteration	p.(His1047Arg)	p.(Glu542Lys)
Affected cells (%)	36	52 (biopsy I) 34 (biopsy II)
Tissue analyzed	Ectopic muscle, dorsum of the left hand	I. Ectopic dorsal interosseous muscle, right side II. Ectopic extensor digitorum muscle, right side
Method for detection	Gene panel dPCR	Whole genome sequencing (WGS) Digital polymerase chain reaction (dPCR)
Description	Unilateral overgrowth of the left arm and forearm muscle, with no signs of edema neither fatty infiltration nor vascular anomaly. Swan neck‐deformity of the index finger and ulnar deviation	Bilateral overgrowth of arms and forearms muscle, with no signs of edema nor fatty infiltration
Ectopic muscles. Localization and characterization. ‐ In hand	‐ Larger fibrotic muscle mass around the extensor tendons on dorsal side of the hand ‐ Transverse ectopic muscle on the dorsal side of the proximal phalanx in the index finger ‐ Normal junctura between second and third extensor indicis communis tendons was replaced by ectopic muscle	Right hand: ‐ Fibrotic and hypertrophic muscle above the first dorsal interosseous muscle attached to the ulnar side of first metacarpal ‐ Hypertrophic abductor pollicis brevis ‐ Six accessory longitudinal muscles attached to the proximal phalanx of digiti two (ulnar), three (ulnar and radial), four (ulnar and radial) and five (radial) originating in separate tendons below the carpal tunnel. Attached to fascia in the forearm ‐ Ectopic abductor digiti minimi muscle ‐ Ectopic short flexor digiti minimi muscle ‐ Broad and extended adductor pollicis muscle inserting in fascia above fifth metacarpal ‐ Palmar aponeuroses and carpal‐ligament replaced by ectopic muscle mass to a high extent
Ectopic muscles. localization and characterization. ‐ In forearm	‐ Ectopic pale fibrotic muscle in volar forearm next to the normal flexor carpi radialis muscle. No wrist or finger movement was noted when traction was applied to the attached tendon	Bilateral in forearms: ‐ Accessory muscle mass originating deep to extensor digiti communis from middle of forearm to insertion in digit two to five via separate broad tendons. Muscle had separate nerve branches. ‐ Accessory extensor pollicis longus (EPL) muscles with tendons parallel to normal EPL Right forearm: ‐ Accessory hypertrophic muscle above the normal brachioradialis muscle ‐ Accessory abductor pollicis longus muscle
Other aberrations	‐Swan‐neck deformity of the left index finger ‐Intrinsic plus position finger two and three ‐Widening between metacarpals	‐ Broad junctura ‐ Missing extensor retinaculum ‐ Missing palmar fascia ‐ Intrinsic plus position finger two and three ‐ Abducted thumbs ‐ Widening between metacarpals
Vascular anomalies	‐	Absent digital volar arteries
Pathology	Increased perimysial and endomysial fibrosis, associated with marked fiber size variability, with scattered hypertrophic fibers and many small fibers. Occasional fibers with rimmed vacuoles were found. There were also rounded eosinophilic fibers.	The pathology differed between the two muscles. In muscle I, variation in muscle fiber size and increase of connective tissue was seen while in muscle II, eosinophilic rounded fibers were present in otherwise rather well‐preserved tissue.

F = female, M = male. The aberrations in patient 2 seemed symmetrical but because of different operations performed on the right and left hand it was not confirmed in all locations.

**Patient 2:** An 18‐year‐old female with normal physical and cognitive development was born as the second child to healthy, non‐consanguineous parents. Pregnancy and delivery were normal; her birth weight was 3300 g and her length was 49 cm. Bilateral symmetric hypertrophic hands, arms and upper part of her trunk were noted already during the neonatal period. During childhood, she was very strong in her arms and hands and could climb using only her upper limbs (Figure 1E). She was referred for hand surgery because the muscular hypertrophy caused functional limitations with difficulties to grip small objects and problems with the pinch grip. Wide hands and abducted thumbs with difficulty to perform the thumb opposition movement were observed. Rotations osteotomy of the first metacarpal and repeated botulinum toxin injections in the hands' intrinsic muscles were performed to improve the pinch grip. The growth was proportional and no progression of the muscular hypertrophy was noted. The difficulty to move the wrists, fingers rotations and elbows deformity led to several surgical procedures. At least 13 ectopic muscles were discovered during surgery (Table 1, Figure 1D, F, G).

### 3.2 Genetic findings

**Patient 1**: In the affected tissue, we identified a mosaic *PIK3CA* hotspot mutation; c.3140A > G, p.(His1047Arg) in 1154 out of 6524 reads (18%) with the oncomine solid tumor panel. The mutation was confirmed and quantified using digital PCR (dPCR), which showed a mutant allelic frequency of 18%, indicating 36% affected cells in the analyzed sample (Table 1).

**Figure 2:**
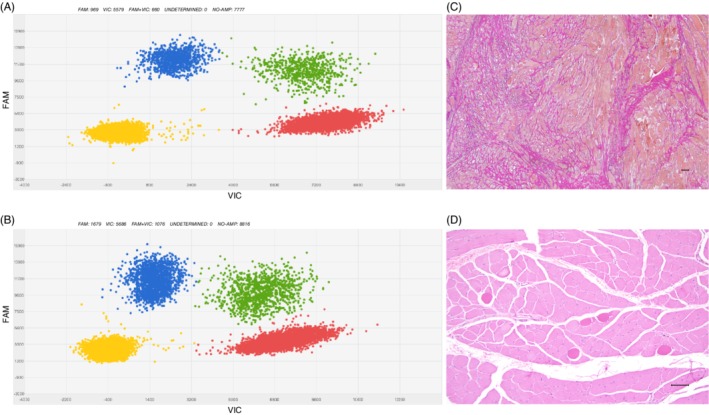
Genetic and pathologic findings in patients. A, B, Representative results from dPCR of DNA from ectopic muscle biopsies. Digital PCR quantifies the load of mosaic mutation, c.1624G > A (p.Glu542Lys), in DNA extracted from ectopic dorsal interosseous muscle and ectopic extensor digitorum muscle from patient 2. Blue cluster (FAM) shows signals from mutant allele, red signals (VIC) from reference allele and green signals from both mutant and reference alleles. Yellow cluster represents the wells where no amplification signal was detected. C**,** Histopathological findings from affected muscle in patient 1. Severe changes with increase in connective tissue (van Gieson). D**,** Histopathological findings from affected muscle patient 2. Scattered rounded eosinophilic muscle fibers in otherwise normal looking tissue (H‐E). Bars: 100 μm

**Patient 2:** WGS identified a mosaic *PIK3CA* hotspot mutation; c.1624G > A, p.(Glu542Lys) in 10 out of 42 reads (24%) in the affected tissue. The mutation was confirmed and quantified using dPCR, which showed a mutant allelic frequency of 26% in muscle biopsy I and 17% in muscle biopsy II, indicating 52%, respectively, 34% affected cells in the analyzed materials (Figure 2A‐B). The mutation was not detected in blood samples from the patient.

### 3.3 Pathological findings

**Patient 1:** Cutting of the unfixed tissue showed muscle with fibrotic streaks. Frozen H‐E stained sections and paraffin sections stained with H‐E and van Gieson showed extensive changes. Great variation in fiber size was seen with scattered hypertrophic fibers and many small fibers. Muscle fiber splitting was present. In some fibers, rimmed vacuole‐like structures were found, which showed immunoreactivity for the antibody p62. A few fibers looked necrotic and some fibers seemed to be regenerating. The number of internalized nuclei was increased. Rounded fibers with increased eosinophilia in H‐E staining were present in the tissue. Inflammatory cells were scarce. In some fascicles, the muscle fibers were separated by excessive endomysial connective tissue and the perimysium was also thickened (Figure 2C). Occasional fascicles had a more normal appearance. Some adipose tissue was present in the material.

**Patient 2:** The pathology was different between the two muscle biopsies I and II (Table 1). In biopsy I, widespread, prominent lesions were found with variation in fiber size and increased number of internalized nuclei. Occasional fibers appeared necrotic and some seemed to be regenerating. A few small aggregates of inflammatory cells were seen. The amount of connective tissue was increased separating the muscle fibers. Some adipose tissue was also found. In regions of more normal appearing muscle, foci of pathologically changed fibers with variation in diameter and an increase of internalized nuclei were noticed together with increased connective tissue. Some rounded eosinophilic fibers could be seen in both muscles with pronounced pathology as well as in otherwise normal tissue. In biopsy II, the pathology consisted mainly of the presence of somewhat rounded eosinophilic muscle fibers. In some fascicles, only one was found, in others they were frequent (Figure 2D). Only occasionally these lesions showed variation in fiber size and an increase in connective tissue.

## 4 DISCUSSION

In this study, we report two individuals with somatic mosaic *PIK3CA* hotspot mutations and a similar phenotype with upper limb muscle overgrowth and ectopic muscles. Patient 1 has unilateral engagement with at least four ectopic muscles in his left upper limb, while patient 2 has a bilateral upper limb overgrowth and at least 13 ectopic muscles. Both patients displayed supernumerary muscles in locations in the upper extremity where there normally should not exist muscles. Therefore, the *PIK3CA* mutated cells seem to have acquired an ability to become muscles when they normally should have differentiated to tendons or fascia. Studies in mouse have shown that activation of mTORC1 signaling in tendons causes impaired collagen fibrillogenesis, disorganized fibers, hypercellularity, and neovascularization^35^ and it is possible that this is similar to what we describe in our two patients. We speculate that our findings indicate that *PIK3CA* has a role in pluripotency and cell fate in early human development.

All vertebrate skeletal muscles, apart from superficial neck muscles, derived from the paraxial mesoderm.^36^ The mesoderm is derived from the primitive streak in the middle of the epiblast plate.^37^ The exact position in the primitive streak decides cell fate.^38^ After invagination, the bilateral paraxial mesoderm, caudal to the head, forms somites which eventually develop into different compartments, such as the sclerotome and the syndetome, which in turn give rise to axial bones, tendons and connective tissues, and the dermomyotome containing proliferating progenitors of all skeletal muscles of the body.^39^, ^40^ The somatic mosaic *PIK3CA* mutation in patient 2 must have occurred at an earlier stage than previous cases that have been reported to occur at day 20 to 56.^4^ Because she has bilateral findings with symptoms involving the trunk and because the somites are located on either side of the midline, the most probable explanation is that the mutation occurred before day 15 post‐fertilization in a cell of the primitive streak in the middle of the embryo just before the process of invagination. During invagination, a group of mutated cells in the primitive streak, predetermined by location to mesoderm, split and migrated to each side of the embryo. In patient 1, who has a unilateral involvement of isolated muscular hypertrophy and supernumerary muscles, mutagenesis must have occurred after day 21 post‐fertilization.

In 2010, Ogino et al described 35 cases with muscular hypertrophy of the hands and/or arms. Similar to our patient 1, 34 out of 35 had unilateral involvement. Similar to our patient 2, one 8‐month‐old male was bilaterally affected. Other common findings were ulnar deviation of the fingers, flexion of the metacarpophalangeal joints, widening of metacarpal spaces, and abducted thumbs. In three operated patients, aberrant accessory muscles were detected but none of these cases were confirmed by molecular analysis. Aberrant accessory muscles have also been reported in other surgically treated patients with upper limb muscle hypertrophy^5^ and in some cases of carpal tunnel syndrome,^41^ but no genetic analysis of aberrant muscles have been performed to rule out somatic mosaic *PIK3CA* mutations. To the best of our knowledge, there are three patients previously described with isolated upper limb muscle overgrowth and with a confirmed *PIK3CA* mutation.^7^, ^8^ In 2014, Castiglioni et al reported the first mosaic *PIK3CA* mutation in a patient with unilateral isolated muscular hypertrophy of the left hand and arm and aberrant muscles. In 2018, al‐Quattan et al reported two additional patients with mosaic *PIK3CA* mutation, isolated muscular overgrowth and hypoplasia of the index finger. In addition, one patient with segmental symmetric overgrowth of the hands, arms, shoulders, and neck, and a lipomatous mass on the upper back, shoulder and neck, has been described.^42^

The somatic mosaic *PIK3CA* mutations c.3140A > G and c.1624G > A in our patients are both hotspot mutations that have previously been detected in neuroectodermal and mesodermal tissue derivatives in PROS patients. The same hotspot mutations are also common in endodermal tissue derivatives in cancer. The reason behind this tissue specificity is unknown.^4^ The endoderm is the innermost of the three germ layers that is formed first. We suggest that early mosaic constitutional mutations in the epiblast might be lethal already during gastrulation and that somatic mutations that occur in differentiated cells are tolerated by the cell but give rise to cancer. A possible explanation could be that *PIK3CA*, depending on cell of origin, switches cell fate in different directions.

Because of the relatively low prevalence, the psychological burden for the families and possible publication bias, it has been debated whether individuals with PROS are at increased risk of Wilms tumor and should be considered for a surveillance program, similar to what is established for Beckwith‐Wiedemann syndrome (OMIM 130650) and hemihyperplasia patients (OMIM 235000).^17^, ^21^, ^25^ There is a possibility that the only patients who are at risk of Wilms tumor are those with a significant burden of renal cells with the activating *PIK3CA* mutation.^27^ If so, dPCR analysis of cell‐free DNA in urine seems promising to identify PROS patients at increased risk of Wilms tumor. However, more studies are needed.

In conclusion, this study adds information about timing of *PIK3CA* mutagenesis during embryogenesis in correlation to phenotype and confirms the diagnostic entity *PIK3CA*‐related muscular overgrowth with ectopic muscles. However, as our study points out, our understanding of *PIK3CA's* role during human embryogenesis is still restricted. Further studies of induced pluripotent stem cells, animal models, and cancer cell lines on the role of somatic mosaic activating *PIK3CA* mutations in cell fate are warranted and could be of great importance for precision medicine in both cancer and PROS.

## CONFLICT OF INTEREST

Nothing to declare.

## Data Availability

Data for this article is available through the corresponding author upon request.
